# Identification of Hub Genes Associated with Immune Infiltration in Cardioembolic Stroke by Whole Blood Transcriptome Analysis

**DOI:** 10.1155/2022/8086991

**Published:** 2022-01-15

**Authors:** Qiaoqiao Li, Xueping Gao, Xueshan Luo, Qingrui Wu, Jintao He, Yang Liu, Yumei Xue, Shulin Wu, Fang Rao

**Affiliations:** ^1^Guangdong Cardiovascular Institute, Guangdong Provincial People's Hospital, Guangdong Academy of Medical Sciences, China; ^2^Research Center of Medical Sciences, Guangdong Provincial People's Hospital, China; ^3^Provincial Key Laboratory of Clinical Pharmacology, Guangdong Academy of Medical Sciences, Guangzhou 510080, China; ^4^Department of Clinical Laboratory Medicine, Southwest Hospital, Third Military Medical University (Army Medical University), Chongqing 400038, China

## Abstract

Cardioembolic stroke (CS) is the most common type of ischemic stroke in the clinic, leading to high morbidity and mortality worldwide. Although many studies have been conducted, the molecular mechanism underlying CS has not been fully grasped. This study was aimed at exploring the molecular mechanism of CS using comprehensive bioinformatics analysis and providing new insights into the pathophysiology of CS. We downloaded the public datasets GSE58294 and GSE16561. Differentially expressed genes (DEGs) were screened via the limma package using R software. CIBERSORT was used to estimate the proportions of 22 immune cells based on the gene expression profiling of CS patients. Using weighted gene correlation network analysis (WGCNA) to cluster the genes into different modules and detect relationships between modules and immune cell types, hub genes were identified based on the intersection of the protein-protein interaction (PPI) network analysis and WGCNA, and their clinical significance was then verified using another independent dataset GSE16561. Totally, 319 genes were identified as DEGs and 5413 genes were clustered into nine modules using WGCNA. The blue module, with the highest correlation coefficient, was identified as the key module associated with stroke, neutrophils, and B cells naïve. Based on the PPI analysis and WGCNA, five genes (*MCEMP1*, *CLEC4D*, *GPR97*, *TSPAN14*, and *FPR2*) were identified as hub genes. Correlation analysis indicated that hub genes had general association with infiltration-related immune cells. ROC analysis also showed they had potential clinical significance. The results were verified using another dataset, which were consistent with our analysis. Five crucial genes determined using integrative bioinformatics analysis might play significant roles in the pathophysiological mechanism in CS and be potential targets for pharmaceutic therapies.

## 1. Introduction

Stroke is a devastating cerebrovascular disease, containing two types: ischemic stroke (IS) and hemorrhagic stroke (HS). Accounting for approximately 80% of all cases, ischemic stroke is the most common subtype, which is triggered by arterial embolization or thromboembolism in the cerebrum [1]. A wide range of clinical manifestations of IS includes physical disability, impaired cognitive and emotional abilities [2].

Constituting about 20% of ischemic stroke, cardioembolic stroke (CS) was mainly caused by nonvalvular atrial fibrillation, myocardial infarction, and rheumatic heart disease [3, 4]. Atrial fibrillation is not only the most common sustained cardiac arrhythmia but also one of the most frequent risk factors that contribute to CS. Moreover, along with fast economic development, social urbanization, aging of population, and changes of lifestyle, the prevalence of CS has increased dramatically, imposing a tremendous medical and social-economic burden on patients. Preventive strategies are generally recommended for all cardioembolic stroke patients, including universal elements of cardiovascular risk factor management such as treatment of diabetes mellitus, blood pressure control, alcohol and tobacco reduction, and antiplatelet medication [5]. Diagnosis of stroke was restricted to history, physical examination, and radiological imaging, which were with limited availability and higher cost. Furthermore, due to the lack of specific early diagnostic markers at the early onset of CS, missed diagnosis and misdiagnosis forces remain relatively common in patients. Thrombolytic therapies are currently the most effective treatment available after CS, regrettably, due to the main method for the treatment of cerebral infarction which is limited by a time window (about 3 h), which results in only around one-third of patients with diagnosed CS which are suitable for receiving these curative therapies [6]. Hence, identification of specific biomarkers for patients at the early onset of CS will prove beneficial. The mechanism of cardioembolic stroke (CS) is complex and involves a myriad of distinct pathogenic factors, consisting of inflammation, oxidative stress, excitotoxicity, apoptosis, excitotoxicity, ion imbalance, and neuroprotection [7]. Nevertheless, the detailed communicative regulatory mechanisms leading to CS remain incompletely understood. Increasing evidence indicates that immune cells play a considerable role in the pathogenesis of CS. Immune-mediated inflammatory markers such as CRP and IL-6 have been reported to be associated with CS [8]. However, the specific molecular mechanism underlying immune or inflammatory marker-mediated CS still needs further investigation.

Weighted gene correlation network analysis (WGCNA) is used to build a coexpression network, detect gene modules, and assess the relationships between gene modules and the biological phenotypes in order to screen the candidate diagnostic biomarkers or potential therapeutic targets.

In our study, we aimed to explore the association between immune cells and CS using integrated bioinformatics methods. CIBERSORT was applied to estimate the proportions of 22 immune cells based on the gene expression profiling of CS patients, and WGCNA was then used to identify the key module associated with CS and immune infiltration. Candidate hub genes were then identified within the key modules. Based on the protein-protein interaction (PPI) network, hub genes were identified. Potential clinical significance of the genes was then determined by using the receiver operating characteristic curve analysis. We hope that this research can offer new insights into significant diagnostic biomarker and potential therapeutic targets for treating CS.

## 2. Methods

### 2.1. Medical Ethics

The raw datasets were available from the NCBI Gene Expression Omnibus repository under accession number (GEO https://www.ncbi.nlm.nih.gov/geo/info/linking.html; GSE58294 and GSE16561). In our study, neither human trials nor animal experiments were applied.

### 2.2. Data Acquisition

We downloaded the corresponding datasets (GSE58294, GSE16561) available from the GEO database for further analysis. In the dataset of GSE58294 (GPL570), the expression matrix of a total of 92 individuals was acquired from the blood samples, including 69 cardioembolic stroke patients and 23 controls. Cardioembolic stroke subjects were analyzed at three time points: less than 3 hours, 5 hours, and 24 hours following the event. The GSE16561 dataset (GPL6883) contains 39 diagnosed with ischemic stroke, and 24 healthy control subjects. All samples were obtained from the blood of patients.

### 2.3. Data Preprocessing and Differentially Expressed Gene (DEG) Screening

The origin microarray data preprocessing, including normalization and background correction, was performed by using the “Affy” package in R; then, the gene expression profile was generated [9]. Principal component analysis was performed to distinguish the cardioembolic stroke and control samples. Differentially expressed genes (DEGs) between two groups were identified using the Bioconductor package Limma (linear models for microarray analysis) [10]. Genes with ∣log2 fold‐change (FC) | >1 and adjust *p* value < 0.05 were regarded as statistically significant DEGs.

### 2.4. GO and KEGG Analyses

Gene Ontology (GO) analysis and a Kyoto Encyclopedia of Genes and Genomes (KEGG) term enrichment analysis were performed using ClusterProfiler software in R language, which showed the biological processes (BPs), cellular components (CCs), molecular functions (MFs), and pathways related to DEGs and genes in the key modules identified in the following. The enrichment significance threshold was set to an adjusted *p* value below 0.05 [11].

### 2.5. GSEA Analysis

Using the median expression level of Mast Cell Expressed Membrane Protein 1(MCEMP1) as cutoff, cardioembolic stroke samples were divided into low and high expression groups. Gene set enrichment analysis (GSEA) was conducted by the “gseaplot2” package to identify the differentially activated signaling pathways in the high MCEMP1 expression group. An ∣NES | >1 and FDR < 0.25 were considered as statistically significant (NES: normalized enrichment score; FDR: false discovery rate) [10].

### 2.6. Immune Cell Infiltration Analysis

Normalized gene expression matrixes were utilized to estimate the relative proportions of 22 types of infiltrating immune cells using the CIBERSORT algorithm [12]. The correlation between immune cells was determined by Spearman's correlation and visualized by heatmap. Next, significant immune cells between cardioembolic stroke and control samples were screened with the threshold Wilcoxon test at *p* value < 0.05.

### 2.7. Weighted Gene Coexpression Network Analysis

#### 2.7.1. Construction of Coexpression Network

The genes ranking in the top 25% of the median absolute deviation in the corresponding expression matrix were selected for Weighted Gene Coexpression Network Analysis (WGCNA) by the “WGCNA” package in R [13]. Briefly, WGCNA was applied to construct a coexpression network based on the matrix of pairwise Pearson correlation coefficients. To satisfy the scale-free topology, an appropriate soft thresholding power *β* should be determined. Then, the genes can be clustered into different functional modules with different colors, which were clustered and classified by the dynamic tree cut algorithm with min. Module size was 50, and the minimum height for merging modules was 0.25. The grey module represented the genes that cannot be merged.

#### 2.7.2. Correlation Analysis and Identification of Key Modules

Module eigengenes (MEs) were considered to be a representation of the corresponding gene expression profile in different modules. Stroke and immune cell infiltration levels were selected as the main clinical traits. The module membership (MM) was defined as the correlation of MEs with gene expression. Gene significance (GS) was defined as the correlation coefficient in the Spearman correlation between gene expression and clinical traits. Modules with the highest GS levels were regarded as key modules and selected for further analysis. Furthermore, genes with MM > 0.8 and GS > 0.5 were defined as hub genes [14].

### 2.8. Construction of PPI (Protein-Protein Interaction) Network

DEGs were imported to the search tool of the STRING database to generate the PPI network identifying the interactions between the hub genes with the threshold of interaction score > 0.9. The hub genes' expression pattern and biological function in the PPI network were visualized by “igraph” (version 1.2.6) and “ggraph” (version 1.0.1) packages in “R” [15, 16]. PPI networks of *MCEMP1* were calculated using the GeneMANIA algorithm [17].

### 2.9. Identification of Key Genes

To screen out the key genes in the development of cardioembolic stroke, we made intersection of hub genes in the WGCNA and PPI as candidate hub genes. Heatmaps of the candidate hub gene expression patterns were generated with the R package “ComplexHeatmap” (version 2.0.0) [18]. In order to ensure the accuracy and robustness of identification of key genes, CytoHubba, a plug-in Cytoscape software (version 3.6.7), was applied to screen the top 10 key genes in candidate hub genes' PPI networks via the degree methods [19]. The intersection of five algorithms in CytoHubba was employed to generate real key genes.

### 2.10. Correlation Analysis of Immune Cells and Key Genes

We investigated the relationship between key gene's expression and relative percentages of immune cells in cardioembolic stroke samples by using Spearman correlation test analysis. The results were visualized using the R package ggplot2 in R software [10]. *p* < 0.05 was considered statistically significant.

### 2.11. Receiver Operating Characteristic (ROC) Analysis

To identify the potential clinical significance of key genes, the diagnostic values of the key genes in GSE58294 were evaluated by applying “pROC” packages [20]. Another dataset (GSE16561) was used for independent verification.

### 2.12. Validation of the Key Gene Expression

We downloaded the validation datasets GSE58294 and GSE16561 from the GEO database (http://www.ncbi.nlm.nih.gov/geo/). All expression values of genes were normalized. A Wilcoxon rank-sum test was performed by comparing the key gene expression value between stroke and control using a *p* value < 0.05 to indicate statistical significance. Box plots of the expression of key genes were illustrated by the ggplot2 R package.

### 2.13. Statistical Analysis

All data analyses were performed in R v3.6.3. Details of these bioinformatics analyses were described in corresponding subsections. A *p* value < 0.05 was defined as statistically significant.

## 3. Results

### 3.1. Data Preprocessing and DEG Screening

**Figure 1:**
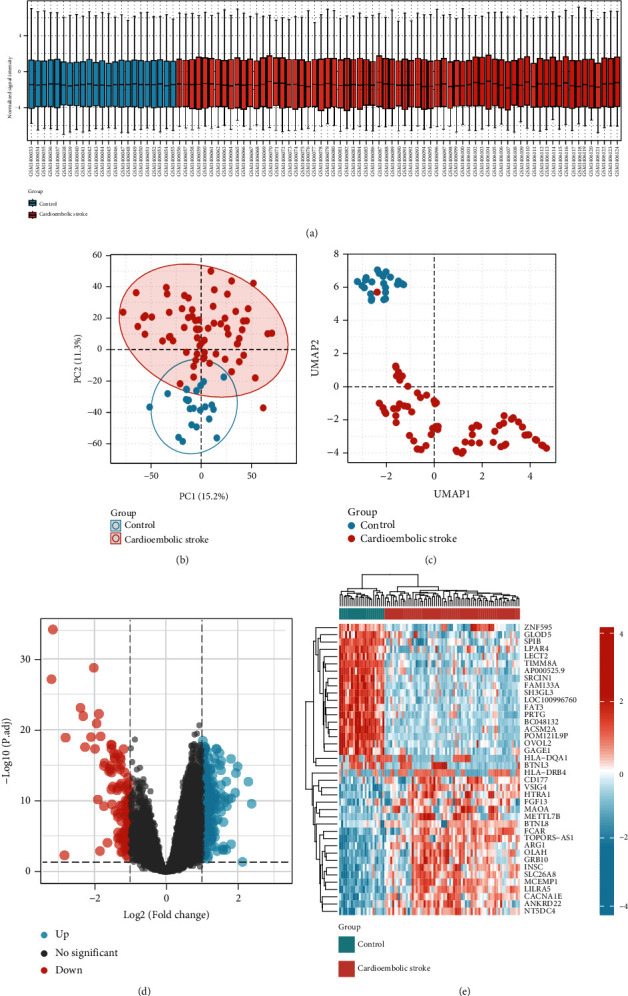
Data preprocessing and identification of DEGs. (a) Box plots after normalization of the raw data between cardioembolic stroke and healthy control samples. (b) PCA for cardioembolic stroke and healthy control samples. (c) UMAP analysis. (d) The volcano plot of DEGs. (e) The heatmap of top 40 DEGs. DEGs: differentially expressed genes; PCA: principal component analysis; UMAP: uniform manifold approximation and projection.

Box plots after normalization of the raw data are illustrated in Figure 1(a). Principal component analysis (PCA) and uniform manifold approximation and projection (UMAP) analysis showed a good distinction between cardioembolic stroke and control samples (Figures 1(b) and 1(c)). Under the screening criteria of *p*adjust < 0.05 and ∣log2 fold‐change (FC) | >1, a total of 319 genes were identified as DEGs, of which 198 genes were upregulated and 121 genes were downregulated. The volcano plot of DEGs is displayed in Figure 1(d). DEGs in GSE58294 were arranged based on the fold change of expression values, the top 40 were illustrated by applying heatmap (Figure 1(e)).

### 3.2. Functional Enrichment Analyses

**Figure 2:**
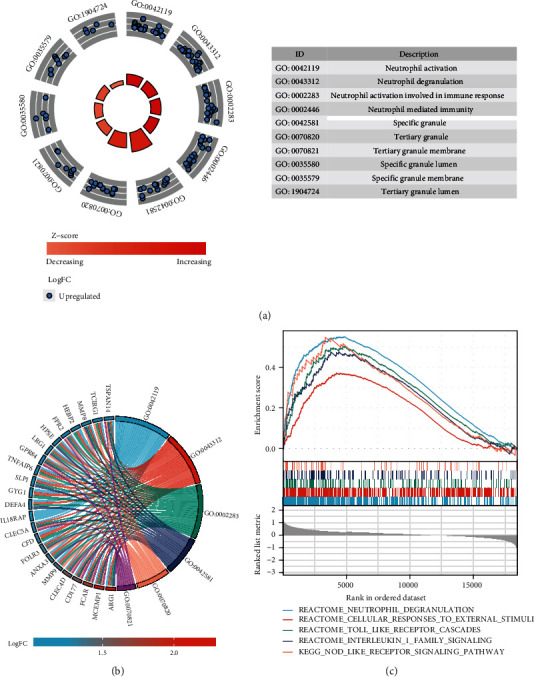
Functional enrichment analysis of DEGs. (a) GOCircle plot showing the top 10 of significantly changed functional terms of DEGs. The height of bars in the inner ring represented the -log 10 adjust P values of GO terms, with higher bars indicating higher significance of the GO category, and color corresponded to the z-score. The scatter plots in the out ring showed the expression levels of each gene (log fold change) in the corresponding GO terms. The description of GO categories was displayed in the table at the bottom. (b) GO chord plot showed genes linked by ribbons to their corresponding GO terms. Different colors represented different GO terms. (c) Results of gene set enrichment analysis in the cardioembolic stroke group.

**Figure 3:**
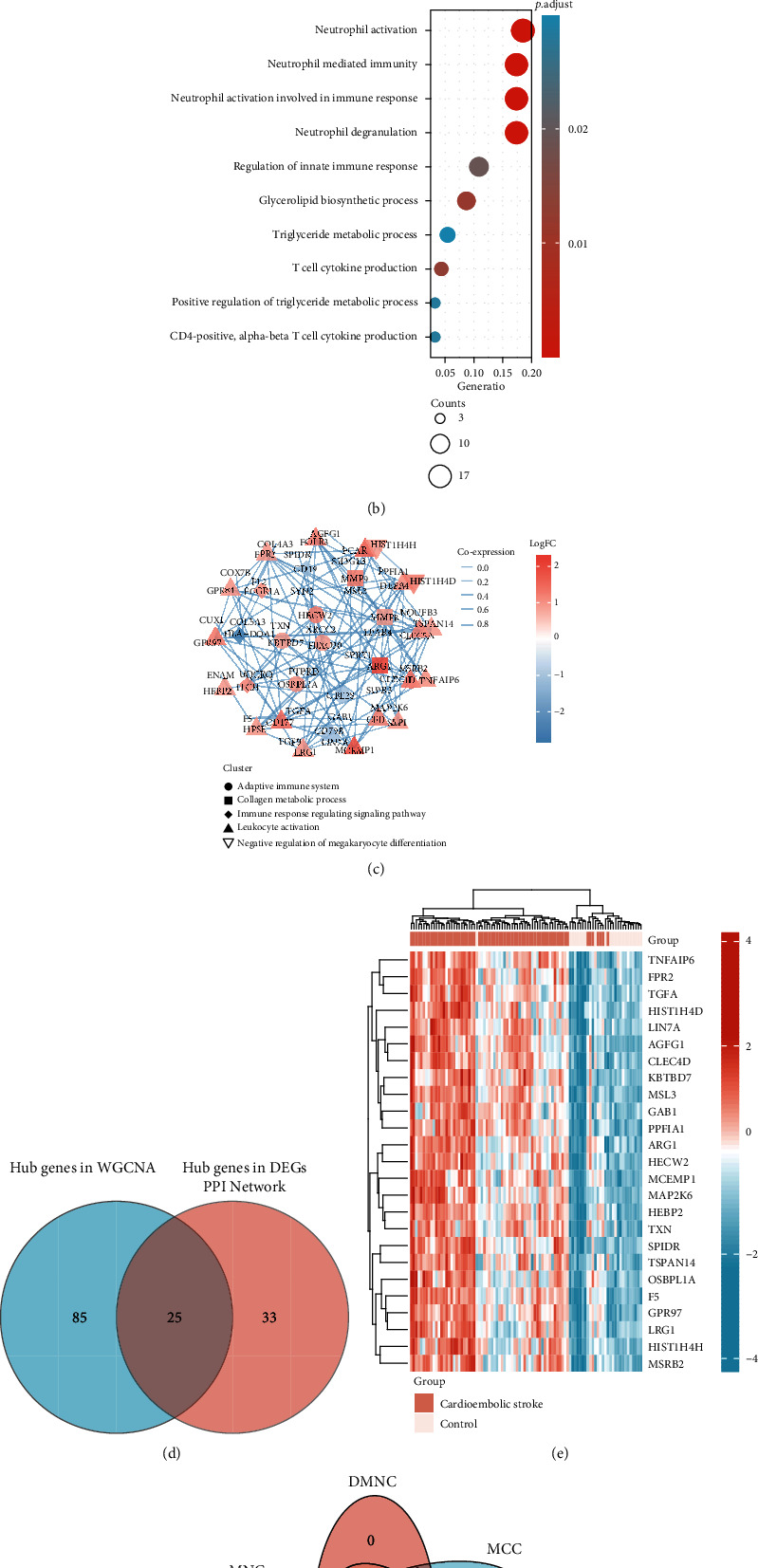
Identification of the key genes. (a) Heatmap of 110 hub genes in blue module. (b) GO analysis of 110 hub genes in blue module. (c) PPI network of 58 DEGs. The red color represented the upregulated genes while the blue color represented downregulated genes. DEGs were subsequently divided into different clusters based on their biological functions. The width of intergenic lines indicated the score of coexpression. (d) Venn plot of hub genes in WGCNA and DEGs. A total of 25 genes were identified as key genes. (e) Heatmap showing the expression profile of 25 key genes. (f) A Venn diagram between five algorithms of CytoHubba. CLEC4D, MCEMP1, GPR97, FPR2, and TSPAN14 were determined as the crucial genes in the cardioembolic stroke.

All DEGs were selected for function enrichment analysis; the top 10 most significant GO terms according to their adjust *p* values were displayed in GOCircle plots (Figure 2(a)). The majority of terms in the biological process category were associated with neutrophil activation (GO:0042119), neutrophil degranulation (GO:0043312), and neutrophil activation involved in immune response (GO:0002283). Genes involved in biological processes that were upregulated in cardioembolic stroke were shown using a chord plot (Figure 2(b)). We further explored our microarray data by using GSEA with the “ggplot2” package in R language; the results indicated that the cardioembolic stroke groups were mostly enriched in terms of neutrophil degranulation, cellular response to external stimuli, toll-like receptor cascades, interleukin-1 family signaling, and nod-like receptor signaling pathway (Figure 2(c)). Hub genes in key module were selected to perform GO enrichment analyses in order to investigate the biological function, as displayed in Figure 3(b).

### 3.3. Immune Cell Infiltration Analysis

**Figure 4:**
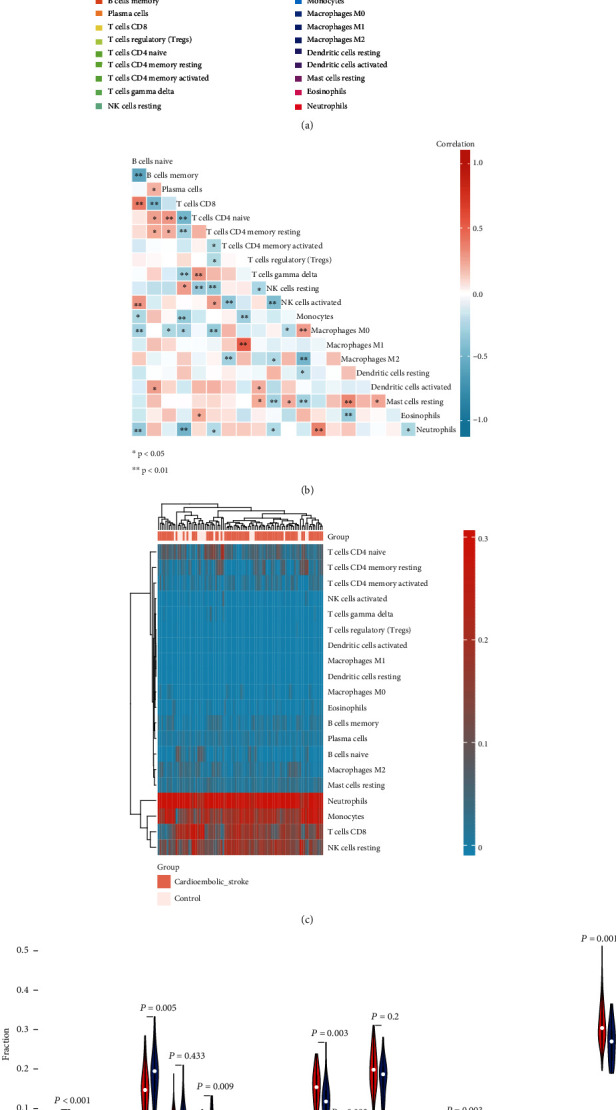
Immune cell infiltration analysis. (a) Relative proportions of 20 types of infiltrated immune cells between cardioembolic stroke and healthy control groups. (b) Correlation heatmap of all 20 immune infiltrated cells. (c) Heatmap of the 20 immune cell proportions in all samples in GSE58294. (d) Violin plot showing the significant changes of the immune infiltration level in cardioembolic stroke compared to the control group.

Applying the CIBERSORT algorithm, we investigated the difference of immune infiltration among cardioembolic stroke and control samples in 22 subpopulations of immune cells (Figure 4(a)). Furthermore, the results of correlation analysis of infiltrated immune cells implied that T cells CD8 and B cells naïve, neutrophils, and macrophages M2 were positively correlated, and T cells CD8 and T cells CD4 naïve, neutrophils, and T cells CD8 were negatively correlated (Figure 4(b)). As shown in the heatmap and violin plot, compared with the control sample, T cells memory activated (*p* < 0.001), NK cells resting (*p* = 0.003), macrophages M0 (*p* = 0.005), macrophages M2 (*p* = 0.003), and neutrophils (*p* = 0.001) infiltrated more in the cardioembolic stroke group, while B cells naïve (*p* < 0.001), T cells CD8 (*p* = 0.005), and T cells CD memory resting (*p* = 0.009) showed the opposite results (Figures 4(c) and 4(d)).

### 3.4. Correlation Analysis between Key Genes and Infiltration-Related Immune Cells

**Figure 5:**
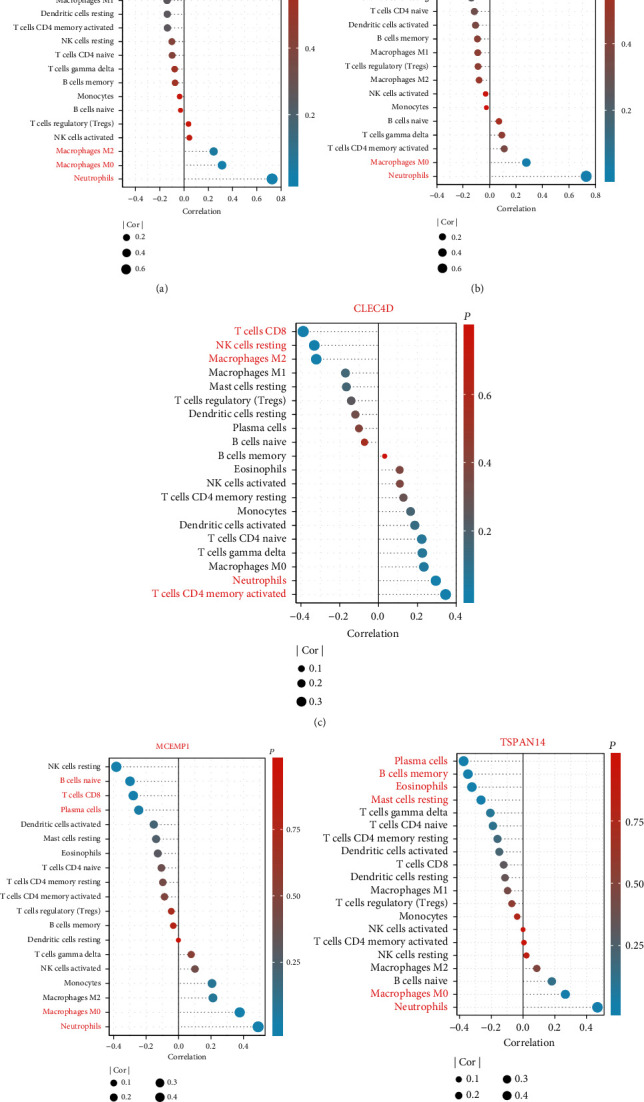
Correlation analysis between key gene expressions and the relative percentages of immune cells in the cardioembolic stroke group. (a–e) Lollipop plots illustrated the relationship between the relative proportions of infiltration-related immune cells and (a) GPR97, (b) FPR2, (c) CLEC4D, (d) MCEMP1, and (e) TSPAN14.

As implied from the correlation analysis, *MCEMP1* displayed a positive correlation with neutrophils (*r* = 0.489, *p* < 0.001) and macrophages M0 (*r* = 0.376, *p* = 0.001) and a negative correlation with NK cells resting (*r* = –0.383, *p* = 0.001). *FPR2* displayed a positive correlation with neutrophils (*r* = 0.728, *p* = 1.26*E* − 12) and a negative correlation with T cells CD8 (*r* = −0.244, *p* = 0.042). *TSPAN14* was positively correlated with neutrophils (*r* = 0.468, *p* < 0.001) and macrophages M0 (*r* = 0.266, *p* = 0.0267). *GPR97* positively correlated with neutrophils (*r* = 0.726, *p* = 1.66*E* − 12) and negatively correlated with T cells CD4 memory resting (*r* = −0.325, *p* = 0.006) and T cells CD8 (*r* = −0.263, *p* = 0.029). *CLEC4D* showed positively correlated with neutrophils (*r* = 0.29, *p* = 0.014) and negatively correlated with macrophages M2 (*r* = −0.321, *p* = 0.007), T cells CD8 (*r* = −0.388, *p* < 0.001), and NK cells resting (*r* = −0.331, *p* = 0.005) (Figures 5(a)–5(e)).

### 3.5. Construction of Coexpression Network

**Figure 6:**
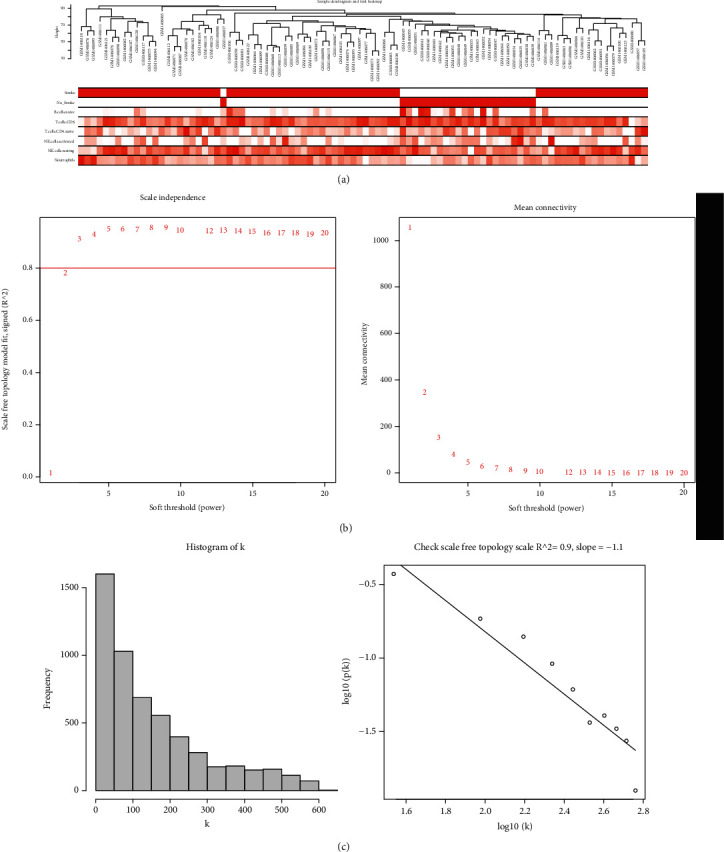
Clustering of samples and selection of the best fit soft-thresholding power. (a) Clustering according to the expression level of cardioembolic stroke patients. The color intensity was proportional to disease status (stroke, no stroke) and infiltration-related immune cells. (b) The cut-off for soft-thresholding power *β* was set to be 0.80, and *β* =3 was determined. (c) The coexpression network exhibits a scale-free topology.

**Figure 7:**
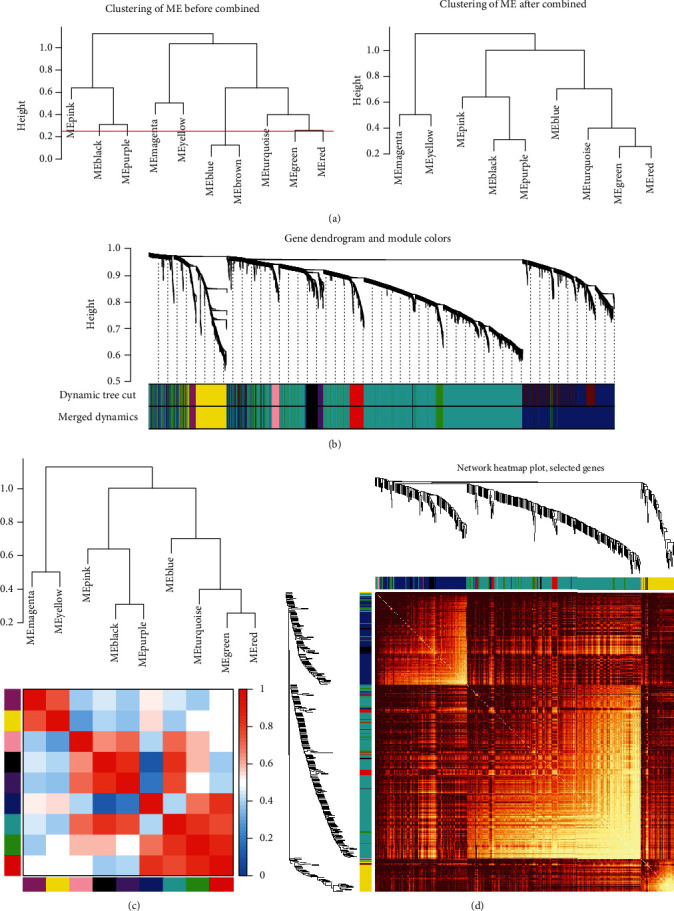
Construction of coexpression network. (a) Clustering dendrograms were cut at a height of 0.25 to detect and combine the similar modules. (b) Origin and merged modules displaying under the clustering tree. (c) Adjacency heatmap of module eigengenes. Red indicated high correlation, and blue represented the opposite results. (d) Clustering dendrogram of nine module eigengenes.

Based on the screening criteria above, a total of 5413 genes were subjected to WGCNA. To detect the possible outlier samples, a cluster tree including 92 samples, clinic traits, and infiltration-related immune cells was performed by applying average linkage methods. Results indicated that no outlier was found in the samples included in the WGCNA analysis (Figure 6(a)). We then established a scale-free (scale-free *R*^2^ = 0.90) coexpression network with the soft-thresholding power *β* = 3 (Figures 6(b) and 6(c)). After merging the highly correlated modules by a clustering height cut-off of 0.25 (Figure 7(a)), nine modules were finally obtained for further analysis. Ultimately, initial modules and merged modules display under the clustering tree (Figure 7(b)). Then, the correlations between the modules were analyzed; there was no significant correlation between different modules (Figure 7(c)). The correlation analysis of transcripts was performed within the modules, and no significant correlation between different modules was detected, implying the reliability of the division of modules (Figure 7(d)).

### 3.6. Identification of the Clinically Significant Modules and Hub Genes

**Figure 8:**
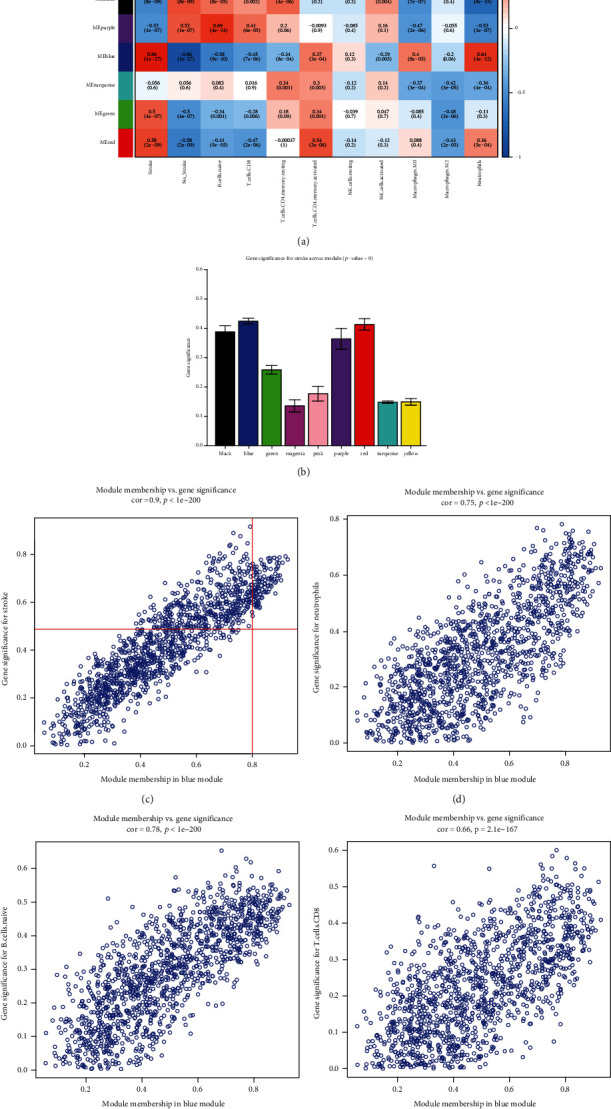
Screening of clinical related modules. (a) Heatmap of module-trait correlation. Red represented positive correlation and blue represented negative correlation. (b) Gene significance for stroke across all modules, the blue module was determined as clinical related module. (c–f) Scatter plot for correlation between the module membership (MM) and gene significance (GS) revised MM and GS (c) stroke, (d) neutrophils, (e) B cells naïve, and (f) T cells CD8.

The association between the modules and clinical traits (disease status and infiltration-related immune cells) was explored by measuring the correlation between ME values and clinical features. The results indicated that blue module was positively correlated with cardioembolic stroke (*r* = 0.86, *p* = 1*e* − 27), neutrophils (*r* = 0.64, *p* = 4*e* − 12), and macrophages M0 (*r* = 0.4, *p* = 8*e* − 05), and negative correlations were observed between blue modules and B cells naive (*r* = −0.58, *p* = 9*e* − 10) and blue modules and T cells CD8 (*r* = −0.45, *p* = 7*e* − 06) (Figure 8(a)). Additionally, the module significance displayed in bar diagram showed the mean gene significance across whole genes of each module. Blue module was identified as the most clinically significant module (Figure 8(b)). Scatter plots of GS for stroke vs. MM (Figure 8(c)), GS for neutrophils vs. MM (Figure 8(d)), GS for B cells naive vs. MM (Figure 8(e)), and GS for T cells CD8 vs. MM (Figure 8(f)) in the blue module were plotted, respectively. The results indicated that blue module was highly correlated with stoke and immune infiltration. Under the screening criteria of ∣MM | >0.8 and ∣GS | >0.5, 110 highly connected hub genes were determined for further analyses.

### 3.7. Screening Key Genes by Integrating Multiple Analysis

Heatmap showed 110 hub genes in the blue module that were highly expressed in the cardioembolic stroke group (Figure 3(a)). To gain further insight into the biological functions of the hub genes in blue module, GO analyses were applied and results indicated they were mainly enriched in “neutrophil activation,” “neutrophil-mediated immunity,” “regulation of immune response,” “T cell cytokine production,” and “CD4 positive, alpha-beta T cell cytokine production” (Figure 3(b)). DEGs were imported into the online tool of the STRING database to generate the PPI network, and a total of 58 genes were identified as hub genes, and their expression pattern and biological functions are also visualized in Figure 3(c). The results implied that most of them were associated with immune response and collagen metabolic process. In addition, we used the Venn diagram to overlap the hub genes in the PPI network and blue module targets and found 25 overlapped DEGs (Figure 3(d)). As displayed in Figure 3(e), the 25 gene expression patterns between stroke and control were also visualized. Five algorithms of the CytoHubba, containing MCC, DMNC, MNC, EPC, and Degree, were then used to process the 25 DEG PPI network to screen the top ten genes. A Venn diagram (Figure 3(f)) was made to build the intersection of genes identified by five algorithms, and *CLEC4D*, *MCEMP1*, *GPR97*, *FPR2*, and *TSPAN14* were determined as key genes.

### 3.8. MCEMP1 and Its Associated Signal Pathways

**Figure 9:**
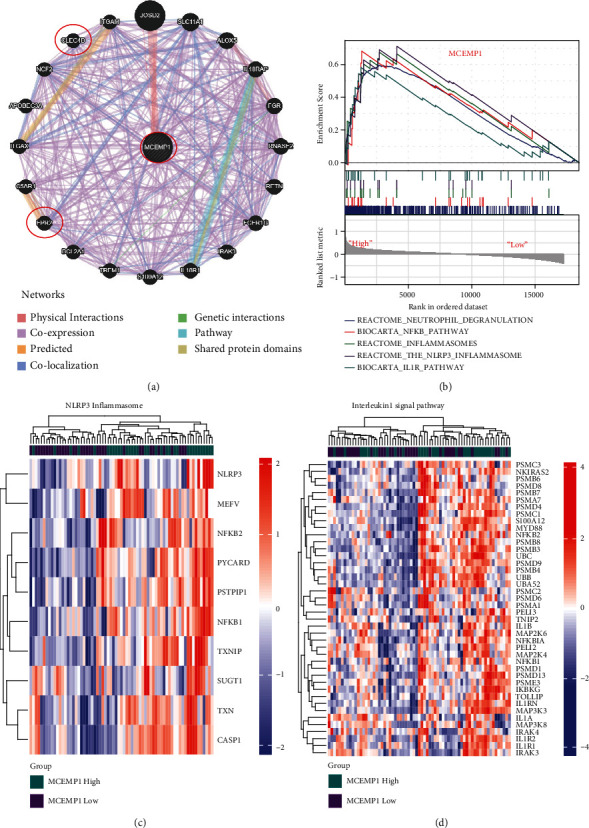
MCEMP1 and its associated signal pathways. (a) MCEMP1 and its coexpression network. (b) GSEA results. (c, d) NLRP3 inflammasome and interleukin-1 signal pathway were significantly enriched in the MECMP1 high group.

We used the full information provided by the Gene-MANIA database to identify the 20 next neighboring proteins of the *MCEMP1*-related query genes; *CLEC4D* and *FPR2* were involved in this analysis. Networks were presented in Figure 9(a). To explore the potential biological functions of *MCEMP1* in CS, GSEA was applied to identify the differentially activated signaling pathways in the high MCEMP1 expression group. Results indicated that the term of neutrophil degranulation, NFKB pathway, inflammasomes, the NLRP3 inflammasome, and IL1R pathway was significantly enriched in the high expression group of *MCEMP1* (Figure 9(b)). Heatmap displayed the associated significantly enriched genes in the term of NLRP3 inflammasome (Figure 9(c)), and interleukin signal pathway (Figure 9(d)).

### 3.9. ROC Analysis of Key Genes

**Figure 10:**
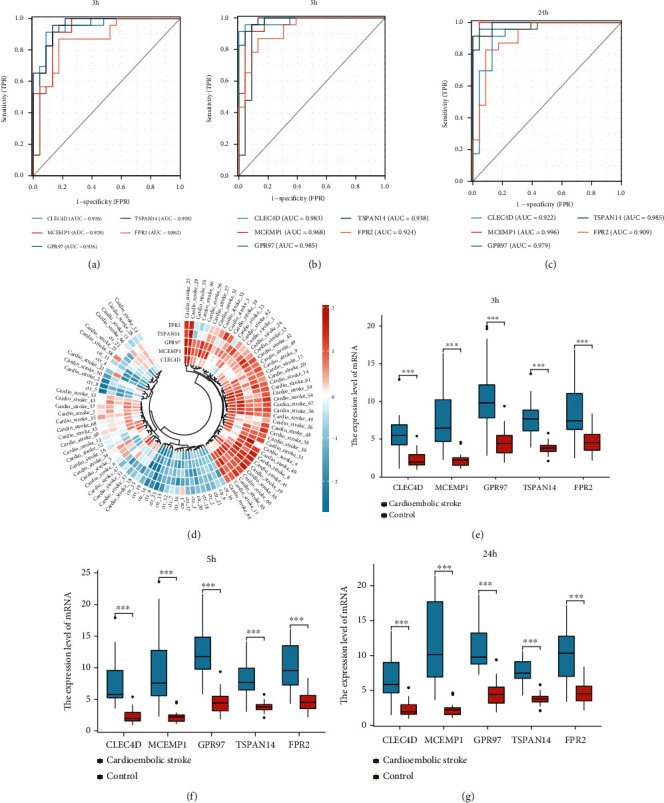
The potential clinical values and expression level of five key genes. (a-c) Applying ROC analysis of 5 key genes to discriminate between cardioembolic stroke and control group, (a) 3 hours, (b) 5 hours, and (c) 24 hours following cardioembolic stroke. (d) Annular heatmap showing the expression of hub genes in each sample. (e–g) Box plots displaying changes in expression levels of CLEC4D, MCEMP1, GPR97, TSPAN14, and FPR2 in 3 h, 5 h, and 24 h after cardioembolic stroke. All crucial genes were significantly increased in patients with cardioembolic stroke compared to normal individuals.

**Figure 11:**
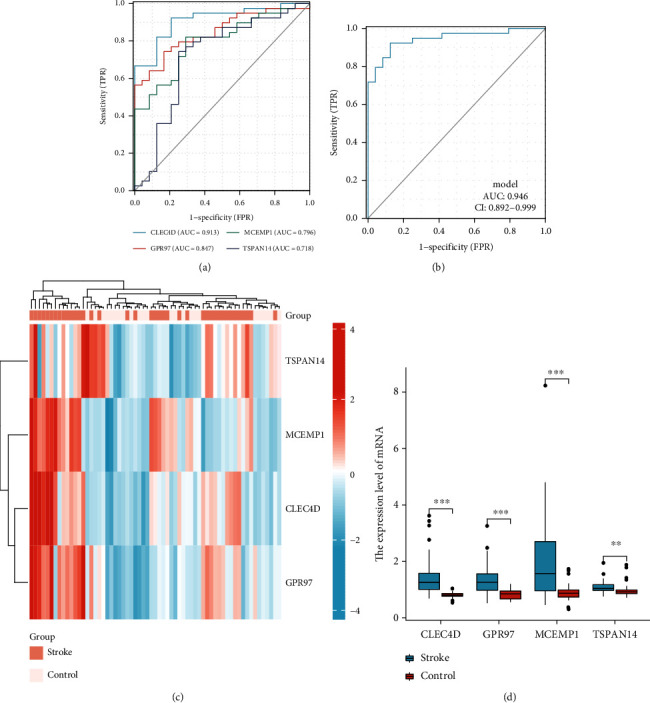
The verification of crucial genes as biomarkers for stroke in an independent dataset. (a) Receiver operating characteristic curves for individual CLEC4D, GPR97, MCEMP1, and TSPAN14 of stroke versus control. (b) Evaluation of clinical diagnostic efficacy of 4 key gene signatures (AUC = 0.946, 95% CI = 0.892–0.999) (Logistic regression model = -14.1075+ 7.9797∗CLEC4D+ 1.8645∗GPR97+ 1.8384∗MCEMP1+ 2.7048∗TSPAN14). (c) Heatmap showing the relative expression levels of CLEC4D, GPR97, MCEMP1, and TSPAN14 in each sample. (d) Box plot indicated the expression of 4 crucial genes following stroke were all significantly higher than healthy individuals.

We performed receiver operating characteristic (ROC) analysis of *CLEC4D*, *MCEMP1*, *GPR97*, *FPR2*, and *TSPAN14* to further validate the diagnostic value of those key genes. The results indicated that all these crucial genes showed potential clinical significance at 3 h (Figure 10(a)), 5 h (Figure 10(b)), and 24 h (Figure 10(c)) following the cardioembolic stroke event. Furthermore, the validation dataset (GSE16561) confirmed the above-presented results: *CLEC4D* (AUC 0.913), *GPR97* (AUC 0.847), *MCEMP1* (AUC 0.796), and *TSPAN14* (AUC 0.718) (Figure 11(c)). To improve the efficiency in distinguishing the capacity of stroke, we constructed the combined diagnosis model of four crucial genes; the AUC value of the stroke reached to 0.946 (95% CI: 0.892–0.999) (Figure 11(b)). These results implied that all crucial genes played key roles in stroke.

### 3.10. Validation of Key Gene Expression

We further validated the expression of these key genes in two datasets. In dataset GSE16561, with the threshold of *p* < 0.05, *CLEC4D*, *GPR97*, *MCEMP1*, and *TSPAN14* were significantly upregulated in the stroke group (Figures 11(c) and 11(d)) (The platform GPL6883 did not explore *FPR2's* expression). In another dataset GSE58294, at 3 h, 5 h, and 24 h postonset, key genes (*CLEC4D*, *MCEMP1*, *GPR97*, *FPR2*, and *TSPAN14*) were significantly upregulated in the stroke group, as compared with those in the normal control (Figures 10(d)–10(g)).

## 4. Discussion

Cardioembolic stroke (CS) results in a high rate of disability, morbidity, and mortality, which is a common central nervous system disease with poor prognosis [21]. CS is a common and complex disease with multiple risk factors and causes, including atrial fibrillation, coronary heart disease, valvular heart disease, hypertension, obesity, and diabetes [21, 22]. Previous studies indicated that inflammation and immunity response were involved in the occurrence and development of CS [23]. Markus et al. have also reported potential cardioembolic stroke biomarkers in their study, including common inflammatory markers CRP, interleukin-6, interleukin-1*β*, and tumor necrosis factor-*α* [24], whereas, currently, there were no specific and highly sensitive biomarkers for distinguishing CS from large stroke cases. Therefore, it is imperative to find potential new candidate biomarkers in order to help physicians to develop a strategy for treating CS at early stages.

In this study, we downloaded the GSE58294 dataset from the available GEO database and estimated the composition of the immune cells using CIBERSORT algorithms based on the expression matrix, then employing WGCNA to determine the modules associated with the immune cell types. Totally, nine modules were screened; among them, blue module was the most significantly associated with CS, neutrophils, B cells naïve, and T cells CD8. To our knowledge, it is the first time to use WGCNA to explore the relationships between immune cell types and CS. We systematically analyzed the proportion of specific types of immune cells in CS patients. It may provide a novel insight into the strategies for diagnosis and immunotherapy of CS. Under the condition of MM > 0.8 and GS > 0.5, 110 candidate hub genes were then identified within the key modules. We then applied functional enrichment analysis on genes, and results indicated that genes were mainly enriched in neutrophil activation, neutrophil-mediated immunity, regulation of innate immune response, and T cell cytokine production. Additionally, DEGs between CS and control were also screened and used to construct the PPI network. Genes within the network were clustered into different subclades, including the adaptive immune system, collagen metabolic process, and immune response regulating signaling pathway. In order to find potential new biomarkers, we generated another new 25 hub genes by taking the intersection of hub genes in DEGs' PPI network and hub genes in key module. Based on the CytoHubba, five hub genes were determined, including *MCEMP1*, *CLEC4D*, *TSPAN14*, GPR97, and *FPR2*. The relationship between hub genes and immune cell was also determined, and results showed that genes were significantly positively correlated with neutrophils and macrophages M0 and negatively correlated with T cells CD8. Finally, by using the ROC analysis, we found that not only individual crucial genes but also the combined diagnosis model of them had potential diagnostic significance.

Mast cell expressed membrane protein 1, this gene encodes a single-pass transmembrane protein MCEMP1. Based on its expression pattern, it is thought to be involved in regulating mast cell differentiation or immune responses. Jian et al. have reported that MCEMP1 was found to be highly expressed in rats with cerebral ischemic stroke [25]. Furthermore, silencing MCEMP1 resulted in the upregulation of vascular endothelial growth factor (VEGF), while downregulation of Caspase3 led to the promotion of microvessel density (MVD) in rats with ischemic stroke [25]. Moreover, silencing of MCEMP1 could increase Ki67-positive cells and reduce terminal deoxynucleotidyl transferase-mediated d-UTP nick end labeling (TUNEL) positive cells in the marginal zone of cortical infarction in rats. Their study has proved that silenced MCEMP1 could suppress neuronal apoptosis and enhance angiogenesis in rats with cerebral ischemic stroke, emphasizing on that MCEMP1 silencing could serve as a therapeutic target for cerebral ischemic stroke treatment. Raman et al. implicated that peripheral blood expression of MCEMP1 within 1 month after stroke has been proposed as a diagnosis and prognostic biomarker for primary stroke [26]. With all this being taken into consideration, MCEMP1 is a key molecule in the regulation and maintenance of the cerebral ischemic stroke. However, the role of MCEMP1 in cardioembolic stroke (CS) and its underlying mechanisms remain poorly understood. Our study was first to associate the immune response and CS and prove that MCEMP1 was correlated with neutrophils, B cells naïve, and T cells CD8. Besides this, NLRP3 inflammasome and interleukin-1 signal pathway were significantly enriched in the MCEMP1 high-expression group. Consequently, all of the results have suggested that the MCEMP1 was involved in the process of inflammatory and immune response and it was worthy of additional investigation and development.

CLEC4D (C-Type Lectin Domain Family 4 Member D), a member of the C-type lectin/C-type lectin-like domain (CTL/CTD) superfamily, acted as a pattern recognition receptor (PRR) of the innate immune system: recognized damage-associated molecular patterns (DAMPs) of pathogen-associated molecular patterns (PAMPs) of bacteria [27, 28]. CLEC4D played vital roles as regulators of cell adhesion, cell-cell signaling, inflammation, and immune response [29]. Moreover, studies have shown that the relative mRNA expression of CLEC4D in peripheral blood of patients suffering ischemic stroke within 24 h after onset was dramatically increased, compared with the normal control group, which was consistent with our analysis results [29]. Additionally, our study indicated that CLEC4D was positively correlated with neutrophils and T cells CD4 memory activated, while negatively associated with T cells CD8, which implied that CLEC4D might act through an inflammatory mechanism dependent upon immune effectors in cardioembolic stroke.

GPR97, also named as ADGRG3, is especially expressed in whole blood, particularly in neutrophils. GPR97 was a significant molecule that regulated the development of B cell and migration of lymphatic endothelial cells in vitro via the small GTPases RhoA and CDC42 [30]. Wang et. al have also verified that GPR97 regulated proinflammatory cytokine production in vitro culture assay and played an important role in the development of experimental autoimmune encephalomyelitis (EAE), which indicated that it may have a therapeutic potential for the treatment of CNS autoimmunity [31]. However, the role of GPR97 in CS is unclear and needs to be further explored.

Tetraspanin 14 (TSPAN14), expressed by many types of tissues, especially whole blood, was involved in neutrophil degranulation, positive regulation of notch signaling pathway, and protein maturation. A previous study has reported that TSPAN14 was correlated with periventricular white matter hyperintensities which was an indicator of a history of cerebrovascular disease [32]. Our results indicated that TSPAN14 was positively associated with macrophages M0 and neutrophils, which suggested TSPAN14 may contribute to CS by participating in immunity and inflammation.

Other genes with high degree in the crucial gene cluster, such as FPR2, also played vital roles in CS pathogenesis. FPR2 is preferentially expressed by monocytes, as previously discussed, and was found to be expressed mainly by mammalian phagocytic leukocytes and involved in inflammation and antibacterial host defense [33]. Vital et al. found that targeting the AnxA1/FPR2/ALX pathway represents an attractive therapeutic strategy for the treatment of thromboinflammation, counteracting, e.g., stroke in high-risk patient cohorts [34]. The findings of Gavins et al. implicated that FPR ligands, particularly in the brain, could be novel and exciting anti-inflammatory therapeutics for the treatment of a variety of clinical conditions, including stroke [35]. There are also several limitations still detected in our present study. First, the data we used was from public databases, which were limited in the sample size. Further research with larger sample sizes should be carried out to validate our results. Second, the functions and potential molecular mechanisms of genes are quite complicated, and further verification of cellular and animal experiments is required.

## 5. Conclusion

In our study, we performed WGCNA to analyze the relationships between immune cell types and cardioembolic stroke (CS) for the first time. Five crucial genes (*MCEMP1*, *TSPAN14*, *CLEC4D*, *GPR97*, and *FPR2*) were identified. These five genes may therefore be potential in CS and are worthy of further investigation.

## Data Availability

The datasets used in this study can be acquired in NCBI datasets (GEO https://www.ncbi.nlm.nih.gov/geo/info/linking.html; GSE58294; GSE16561).
